# Benefits of fermented synbiotic soymilk containing *Lactobacillus acidophilus*, *Bifidobacterium lactis,* and inulin towards lead toxicity alleviation

**DOI:** 10.1016/j.heliyon.2023.e17518

**Published:** 2023-06-23

**Authors:** Maryamsadat Riasatian, Seyed Mohammad Mazloomi, Afsane Ahmadi, Zahra Derakhshan, Saeed Rajabi

**Affiliations:** a Department of Community Nutrition, School of Nutrition and Food Sciences, Shiraz University of Medical Sciences, Shiraz, Iran; b Nutrition Research Center, School of Nutrition and Food Sciences, Department of Clinical Nutrition, Shiraz University of Medical Sciences, Shiraz, Iran; c Research Center for Health Sciences, Department of Environmental Health, School of Health, Shiraz University of Medical Sciences, Shiraz, Iran; d Department of Environmental Health Engineering, School of Health, Shiraz University of Medical Sciences, Shiraz, Iran; e Student Research Committee, School of Health, Shiraz University of Medical Sciences, Shiraz, Iran

**Keywords:** Lead, Soybean milk, Probiotic bacteria, Prebiotic, Oxidative stress

## Abstract

This study aims to evaluate the effect of fermented synbiotic soy milk containing Lactobacillus acidophilus, Bifidobacterium lactis, and inulin on a series of hematological and oxidative stress parameters, as well as serum lead levels in rats. In this study, 56 male Sprague-Dawley rats were randomly assigned to assess probiotics (L. acidophilus or B. lactis), probiotics with prebiotics (i.e., inulin), and the corresponding controls. Several hematologic parameters (red blood cell (RBC)), hematocrit (HCT) and hemoglobin (Hgb)), serum lead levels, superoxide dismutase (SOD) activity, and malondialdehyde (MDA) presence was measured to evaluate changes on day 42. Although a significant difference was observed in serum lead levels, there were no significant changes in hematological and oxidative stress parameters between the study groups. In conclusion, this study demonstrates that administering synbiotic fermented soy milk containing the probiotic Lactobacillus acidophilus and the prebiotic inulin may significantly improve serum lead levels in rats.

## 1 Introduction

Lead (Pb) is a toxic heavy metal with no known physiological role in the body, yet exposure to it has been identified as a global public health concern due to its adverse health effects [[1], [2], [3], [4], [5], [6]]. According to the Advisory Committee on Childhood Lead Poisoning Prevention (ACCLPP) (2012), a lead blood level of 5 μg per deciliter (μg/dL) is the upper reference range value for children and is used as an advisory level for environmental and educational interventions. In May 2021, the Lead Exposure Prevention Advisory Committee, based on information from the National Health and Nutrition Examination Survey (NHANES) recommended lowering the blood lead reference value (BLRV) from 5 μg/dL to 3.5 μg/dL for children ages 1 to 5 years [7]. The FDA and the CDC recommend an Interim Reference Level of 12.5 μg/day for adults [8]. Studies have revealed that even lower levels of lead (Pb) concentration thresholds can have adverse effects, such as cognitive, neurobehavioral, and neurophysiological disorders, as well as impairment of learning ability and intelligence, especially in children [7,[9], [10], [11]]. Lead poisoning can also induce oxidative stress, leading to alteration in gene expression associated with reactive oxygen species (ROS), resulting in serious, irreversible damage to the gastrointestinal (GI), hematological, reproductive, immunological, renal, and hepatic systems [[12], [13], [14], [15]]. Respiratory and gastrointestinal systems are particularly vulnerable to Pb exposure, with children absorbing 40–50% of ingested Pb through the intestine, compared to 10–15% in adults [8].

Besides, the dietary composition and potential nutritional deficiencies can have a significant impact on lead absorption. Specifically, it has been shown that a low-fat and protein-rich diet can reduce Pb absorption, while calcium, iron, zinc, and vitamin D deficiency increases the tendency for Pb absorption in the intestines [2,16]. Currently, conventional technologies for removing heavy metals from the environment are expensive and ineffective when the heavy metal concentration is low [17]. Chelation therapy is the most common therapeutic strategy for increasing heavy metal excretion and treating human poisoning [[18], [19], [20]]; however, it has certain drawbacks in terms of preservation methods, side effects, and safety. Therefore, developing new dietary treatment strategies is essential for avoiding damage caused by lead exposure [20].

The application of dietary probiotics is a novel strategy to safely remove heavy metals from the gut, known as “Gut Remediation” [21]. Probiotics are live microorganisms that have been shown to have many health benefits in humans, including improving gastrointestinal (GI) disturbances, inflammation, and oxidative stress, as well as helping to maintain a healthy intestinal microbial balance [22,23]. *Lactobacillus* and *Bifidobacteria* are part of the healthy GI tract microflora and are found in fermented foods [24,25]. These probiotic bacteria boast maximum antioxidant properties among gut microbes and have been observed to have a remarkable ability to detoxify the human gut from heavy metals, such as cadmium and lead in laboratory settings [25]. *Lactobacillus* and *Bifidobacteria* are two common species of probiotic bacteria that have been found to have beneficial properties for detoxification and gut remediation. Studies have demonstrated their ability to excrete heavy metals and bind to other toxic compounds, such as cyano-toxins and dietary mutagens [[26], [27], [28], [29]]. Moreover, *Bifidobacteria* are part of breast milk and are known to promote the immune system, lower cholesterol levels, and produce B vitamins. Thus, introducing these probiotics into the diet can provide an effective and safe strategy for gut remediation and need to be comprehensively evaluated [21].

Prebiotics, such as inulin, can enhance probiotics growth in the colon and are commonly consumed with probiotics as part of fermented foods, such as in yogurt, soy yogurt, and soymilk, or as dietary supplements [30]. They can be used to prepare soy milk mixture of living microorganisms and substrates that are selectively used by host microorganisms and are beneficial to host health, called synbiotics. This strategy is defined as complementary or synergistic synbiotics. A synergistic synbiotic contains a substrate that is selectively utilized by the co-administered live microorganism. Soy milk provides an excellent alternative to dairy milk, especially for those with lactose intolerance. It is an aqueous extract of soybeans, composed of high-quality proteins and dietary fiber, with no lactose. Soybeans also contain isoflavones that provide powerful antioxidant protection, helping to prevent chronic illnesses such as cancer, cardiovascular disease, osteoporosis, menopausal symptoms, and diabetes mellitus [[31], [32], [33]]. Despite its many benefits, soy milk has some drawbacks, such as its unpleasant taste, and the risk of stomachache or bruising [31,32].

Due to the beneficial effects of probiotics in heavy metal removal and since soymilk, with its beneficial impact on health, can be a proper choice for probiotics introduction (as a fermented food), this study is designed to investigate the effects of fermented symbiotic soy milk containing Lactobacillus acidophilus, Bifidobacterium lactis and inulin against Pb toxicity in rats.

## 2 Materials and methods

Animal experiments were approved and conducted in conformity with the Shiraz University of Medical Sciences Ethics Committee for the care and use of laboratory animals (Ethic No.: IR.SUMS.REC.1392.S6867).

### 2.1 Study design

A randomized controlled study of 7 groups of rats was employed to assess the effect of probiotic bacteria alone (*Lactobacillus acidophilus* or *Bifidobacterium lactis*) or their combined administration with the prebiotic inulin. Lead-contaminated water was provided over 42 days [34]. To evaluate treatments for each group of rats, at day 42, the red blood cell count (RBC), hematocrit (HCT), hemoglobin (Hgb), serum lead levels, superoxide dismutase (SOD) activity, and malondialdehyde (MDA) presence were measured. Finally, evaluating the findings allows us to formulate a proposition on the contents of fermented synbiotic soy milk that could be used as a lead chelator.

### 2.2 Animals

Fifty-six male Sprague-Dawley rats weighing 150–200 g were purchased from the Shiraz University of Medical Sciences. The animals were kept in polypropylene cages under standard conditions (12 h light/dark cycle, constant temperature of 25 ± 2 °C, humidity 50 ± 5%) with *ad* libitum access to standard food (Pars Dam Co., Tehran, Iran) and water.

### 2.3 Lead-contaminated water preparation

While the control group received normal water, the Pb-exposed group received 100 ppm Pb acetate (Merck, Germany) [34]. This contaminated water was prepared using a sensitive scale weighing 0.05 g of Pb acetate. Freshly prepared water containing Pb acetate was served to the animals thrice a week.

### 2.4 Synbiotic fermented soymilk preparation

This study produced four types of probiotics and synbiotic fermented soy milk containing two bacteria (*Lactobacillus acidophilus* and *Bifidobacterium lactis*). Inulin was used for the production of synbiotic soy milk. For this purpose, soybeans were weighed and soaked overnight in distilled water. The water was replaced, soaked soybeans were introduced to distilled water ten times their initial weight, and the mixture was homogenized in a blender for 3 min. After passing the mixture from a filter, the final extract was divided into five equal parts. The 2% (w/w) inulin (Orafti HP, England) was added to two samples and mixed until completely dissolved. Then, all samples were pasteurized at 90 °C for 15 min and cooled to 37 °C. 0.1 g/L of Bifidobacterium lactis or Lactobacillus acidophilus (Christin Hansen, Denmark) was spiked to inulin samples and two other soy milk samples. All the samples were kept in an incubator at 37 °C until the pH reached 4.7 [32]. Fermented soymilk was produced weekly under sterile, hygienic conditions, and probiotic bacteria were manually counted in anaerobic conditions on MRS agar (Liofilchem, Italy) and incubated at 37 °C for 72 h.

### 2.5 Groups

After two weeks of acclimatization, the rats (n = 8) were randomly assigned to 7 groups and marked their tails for identification as follows:

Group I: Normal diet + normal water (negative control), Group II: Normal diet + water containing Pb (positive control), Group III: Normal diet + water containing Pb + soymilk, Group IV: Normal diet + water containing Pb + probiotic soymilk fermented with L. acidophilus, Group V: Normal diet + water containing Pb + synbiotic soymilk fermented with L. acidophilus and inulin, Group VI: Normal diet + water containing Pb + probiotic soymilk with B. lactis and

Group VII: Normal diet + water containing Pb + synbiotic soymilk fermented with B. lactis and inulin.

In order to determine the amount of soy milk required by the rats, the animal's weight was measured weekly using a benchtop scale. The study groups received 10 mL/kg of soymilk, probiotic soybean milk, or synbiotic soymilk once a day for 42 days.

At the end of the study, blood samples were prepared to measure the indicators. Due to having a negative control group, blood samples, and measurements were not performed before the start of the study.

### 2.6 Blood samples

After overnight fasting, rats were anesthetized with an intraperitoneal injection of a Ketamine/Xylazine cocktail (6.6/0.3 mg/kg). Blood samples were obtained from cardiac puncture and divided into two parts natural test tubes to harvest plasma and serum and the other in whole blood in heparin-containing tubes. The test tubes containing blood samples were centrifuged at 3000 rpm for 15 min at 4 °C, and the serum was separated and stored at −80 °C. The serum was used to determine the amount of Pb and malondialdehyde (MDA). Whole blood heparin tubes were used to measure red blood cells, hematocrit, hemoglobin, and superoxide dismutase in red blood cells. The hematologic parameters were measured by Cell Counter (Nihonkohden, Japan) in the Nutrition Research Laboratory.

### 2.7 MDA assessment

Serum MDA concentration (μm/L) was determined according to Zal et al. by measuring thiobarbituric acid reactive substances (TBARS) by spectrophotometric assay (Spectrophotometry, Apel, Japan) [35].

### 2.8 Superoxide dismutase assessment

A commercial kit (Biorex, Iran) measured superoxide dismutase (SOD) activity in erythrocytes. SOD levels were recorded at 505 nm through a standard curve and expressed as unit SOD/mL whole blood (according to the instruction given in the Kit manual).

### 2.9 Lead assessment in serum

All collected samples were digested in UV digestive system using perchloric acid and nitric acid to determine the lead level in the serum. The digested bright yellow solutions were then separately injected into atomic absorption spectrometry (Chem. Theck model CTA 3000, England).

### 2.10 Statistical analysis

All statistical analyses were performed using SPSS software version 21.0 (SPSS Inc, Chicago IL, USA.). Data are represented as the mean ± SD. The data were analyzed using one-way ANOVA followed by *post hoc* Tukey tests for comparisons between multiple groups. Repeated measure ANOVA was also used where appropriate and P < 0.05 was considered statistically significant.

## 3 Results

### 3.1 Microbial culture

Probiotic dairy products should contain at least $10^{6}$-$10^{7}$ CFU/mL of these bacteria. Thus, during the production of products, to ensure the presence of sufficient bacteria, a bacteria culture was prepared and measured from each product. Table 1 shows the average probiotic counts in 6 weeks. Determining the bacterial load confirmed their propagation at adequate levels for their use.

**Table 1:** The amounts of bacteria used in this study (CFU/g).

	Probiotic soymilk (*Lactobacillus)*	Synbiotic soymilk (*Lactobacilus + Inulin)*	Probiotic soymilk (*Bifidobacter*)	Synbiotics soymilk (*Bifidobacter + Inulin)*
Bacterial content	6.2 × 10^7^	19.1 × 10^7^	8 × 10^7^	23.8 × 10^7^

Note; CFU: colony forming unit.

### 3.2 Rats' body weight

The goal of weekly weighing was to determine the gavage content of soy milk based on weight. Table 2 shows the rat's body weight average based on weeks of study in different groups. A gradual body weight increase was observed over six weeks, so the amount of soymilk changed from 1.7 mL in the first week to 2.7 mL in the sixth week.

**Table 2:** Rats body weight average (gr/week).

Groups	First week	Second week	Third week	Fourth week	Fifth week	Sixth week
Control (n=8)	177.75	201.4	233.5	236.7	264.7	275.3
Soymilk (n=8)	174.6	196.58	229.5	245.5	268.5	270
Probiotic soymilk (*Bifidobacter*) (n=8)	172.8	200.2	227.25	254.5	261.5	276
Synbiotics soymilk (*Bifidobacter + Inulin* (n=8)	172.28	196.75	213.4	236.75	240.6	257.9
Probiotic soymilk (*Lactobacillus)* (n=8)	172.95	202.25	221.05	239.95	250	259.1
Synbiotic soymilk (*Lactobacilus + Inulin)* (n=8)	172.8	211.48	228.9	250.1	260	275.7
Lead contaminated water (n=8)	174.6	204.65	220	240.05	251.3	267.9
Total*	173.9 ± 1.90	201.9 + 5.12	224.8 + 6.92	243.3 + 6.88	256.6 + 9.75	268.8 + 7.71

Note:* values for continuous variables, as mean ± SD.

### 3.3 Hematological parameters

Table 3 shows the mean ± standard deviation of hematological parameters (red blood cells, hematocrit, and hemoglobin) in the studied groups. The data shows that the amount of hemoglobin and percentage of hematocrit was the lowest in the soy milk group (13.7 ± 3.50 gr/dL,41.2 + 9.70%) compared to the other groups, while the highest mean was observed in the lead-contaminated water group (16 ± 0.54gr/dL,47.35 ± 4.95%). However, there was no significant difference between the groups (P = 0.26 and P = 0.56 for hemoglobin and hematocrit, respectively). Red blood cells had the highest mean in the rats consuming Bifidobacterium soy milk (8.25 ± 0.75 **10⁶/Ul**), and the lowest in the Lactobacillus group (7.15 + 1.40 **10⁶/Ul**). Again, there was no significant difference between the groups. These findings indicate that treatment with probiotic and synbiotic soy milk products does not cause significant changes in hematological parameters.

**Table 3:** The mean of blood indicators in the groups.

	Hemoglobin (gr/dL)	Hematocrit (%)	Red blood cells (10⁶/Ul)
Control	14.78 ± 1.67	44.75 ± 7.10	7.65 ± 0.60
Soymilk	13.7 ± 3.50	41.2 ± 9.70	7.18 ± 1.80
Probiotic soymilk (Bifidobacter)	15.3 ± 1.30	46.3 ± 8.20	8.25 ± 0.75
Synbiotic soymilk(Bifidobacter + Inulin)	14.65 ± 2.35	43.3 ± 7.25	7.52 ± 1.10
Probiotic soymilk (Lactobacillus)	14 ± 2.90	41.25 ± 9.60	7.15 ± 1.40
Synbiotic soymilk (Lactobacilus + Inulin)	15.5 ± 1.30	44.4 ± 6.80	8.08 ± 0.50
Lead contaminated water	16 ± 0.54	47.35 ± 4.95	8.15 ± 0.33
*P*-value	0.26	0.56	0.12

Note: values for continuous variables, as mean ± SD. Groups are compared using one-way ANOVA. P < 0.05 is considered significant.

### 3.4 Oxidative stress indicators and serum lead level

Table 4 demonstrates the mean and standard deviation of erythrocyte superoxide dismutase (SOD) activity, serum malondialdehyde (MDA), and lead amount in the studied groups. Rats receiving lead-contaminated water showed the highest SOD activity (236.45 ± 118.60). The lowest level of MDA was seen in rats who received simple soymilk (0.08 ± 0.04 μm/L). There was no significant difference between the groups on their erythrocyte SOD activity (P = 0.53) and serum MDA (P = 0.47). These results indicate that soy milk products do not cause a significant change in oxidative stress indicators in rats exposed to lead compared to the control group. Therefore, our results suggest that synbiotic soy milk does not significantly alter erythrocyte SOD activity and serum MDA in rats exposed to lead.

**Table 4:** The mean of MDA, SOD, and lead in the groups.

	MDA (μm/L)	SOD $\left(\frac{unit\;SOD}{ml\;whole\;blood}\right)$	Lead (ppb)
Control	0.11 ± 0.02	199.00 ± 85.05	6.62 ± 2.80 ^a^
Lead contaminated water	0.09 ± 0.02	236.45 ± 118.60	4.15 ± 1.60 ^a, c^
Soymilk	0.08 ± 0.04	217.45 ± 91.05	7.04 ± 3.02 ^b, c^
Probiotic soymilk (*Lactobacillus)*	0.10 ± 0.02	213.35 ± 51.90	5.60 ± 2.20
Synbiotic soymilk (*Lactobacilus + Inulin)*	0.92 ± 0.03	159.45 ± 67.30	3.75 ± 1.20 ^a,b^
Probiotic soymilk (*Bifidobacter*)	0.17 ± 0.23	219.90 ± 12.50	5.09 ± 1.58
Synbiotics soymilk (*Bifidobacter + Inulin)*	0.11 ± 0.04	229.78 ± 112.65	5.62 ± 1.98
P value*	0.47	0.53	0.02

**SOD**: Superoxide dismutase, **MDA:** Malondialdehyde, **ppb**: part per billion, μm/L: micromoles per liter. Note: values for continuous variables, as mean ± SD. Groups are compared using one-way ANOVA. P < 0.05 is considered significant. ^a^ Significant difference between Control group with lead contaminated water and synbiotics (*Lactobacillus*) groups. ^b^ Significant difference between synbiotics(*Lactobacillus*) and soymilk groups. ^c^ Significant difference between lead contaminated water and soymilk groups. **Bold** numbers demonstrate significant *P*-value.

The results of this study show that there is a significant difference between the lead levels of the different groups (P = 0.02). The Lactobacillus synbiotic group had the lowest lead levels (3.75 ± 1.20 ppb), while the soy milk group had the highest (7.04 ± 3.02 ppb). The differences between the control group (6.62 ± 2.80 ppb), lead-contaminated water group (4.15 ± 1.60 ppb), and the synbiotic (Lactobacillus) group were significant. Furthermore, the lead levels of the soy milk group were significantly higher than those of the synbiotic (Lactobacillus) group. Thus, this study demonstrates that synbiotic soy milk containing lactobacillus can significantly reduce blood lead levels in rats exposed to lead compared to the controls and simple soy milk groups.

## 4 Discussion

The present study evaluated the use of synbiotic soybean milk as a detoxification food to reduce the harmful effects of lead entering the bio-cycle and accumulating in the food chain. The results showed that the lowest serum lead level was found in the group that received synbiotic soybean milk with *L. acidophilus* and inulin, indicating that this combination had the greatest effect on reducing lead levels. This finding is consistent with previous in vitro studies [12,25,[36], [37], [38]]. Previous studies have shown that Lactobacillus species can scavenge peroxide, superoxide, and hydroxyl free radicals [27]. Our results, however, showed that *B. lactis* presence did not significantly affect lead levels, contrary to *L. acidophilus*. Therefore, *L. acidophilus* synbiotic soybean milk may be an effective detoxification food for living organisms and can help reduce the harmful effects of lead in the environment.

The proposed mechanism in this in vitro study includes complex formation, ion exchange, absorption, forging, and particle deposition [26]. As gram-positive bacteria, lactic acid bacteria have a thick layer of peptidoglycan, taconic acid, protein, polysaccharide containing carboxyl groups, hydroxyl, and phosphate, which attach to cationic ions, such as Cd and Pb. In addition, it has been shown that gram-positive bacteria have an overall negative charge, which can bind to positively-charged ions. Furthermore, L. acidophilus and B. lactis, as part of the gut microflora, are classified as harmless live microorganisms that provide a health benefit to the host and are an appropriate option for the biological elimination of the toxins [20,25,27,28,36]). There is compelling evidence suggesting that probiotics can help reduce the absorption of heavy metals, in the intestinal tract via mechanisms such as the enhancement of intestinal heavy metals sequestration, detoxification of heavy metals in the gut, altering the transport metal protein's expression, and maintaining the gut barrier function [39].

This study shows a disparity in the efficacy of microorganisms and thus indicates that the process of lead detoxification by probiotics in rats’ GI may be affected by various factors. Numerous studies have shown that the binding and removal of metals by bacteria is a pH-dependent process, which often increases linearly with increasing pH from 4 to 7. Therefore, low pH and the presence of other ions in the digestive system may interfere with the function of probiotics to reduce the serum concentration of lead in rats and justify their low effectiveness in the present study [25,36].

Despite the different fermented synbiotic soymilk versions administered, there was no significant difference in serum hemoglobin (Hgb), hematocrit, and red blood cell (RBC) levels between the groups in the present study. Patil et al. also found no significant difference in RBC counts in battery factory workers with the control group [40]. In our study, the results of RBCs in the group receiving Bifidobacterium lactis probiotic soymilk were consistent with the results of Hsiao et al. [41]. This group, while had one of the highest serum lead levels, had high RBCs levels compared to other groups, but these differences were not significant; In the Hsiao et al. study, due to the inhibition of Hgb production by increasing blood Pb levels and pH production, along with increasing RBC production, the amount of Hgb remained constant during the study period [41,42]. According to this theory, the rate of Hgb was similar to RBC, thus confirming Hsiao et al. theory of dynamic effects of Pb and bone levels and the alignment of Hgb production with RBCs [41]. Hande et al. also confirmed Chun Yin's theory and reported that the RBC levels of exposed workers were higher than the control group [18]. Studies showed that blood Pb reduces the formation of red blood cells by binding to red blood cells, induction of unsaturated fatty acids' peroxidation, and altering the composition of membrane proteins [[42], [43], [44]]. The delta-aminolevulinic acid (δ-ALA) enzyme in the heme biosynthesis cycle links two molecules of ALA by binding to the thiol group and generates the porphobilinogen (PBG). Pb is attached to the sulfide group of these enzymes due to its tendency to bind to thiol molecules, and the ALA molecule accumulates by stopping the heme synthesis. Then, Pb catalyzes the accumulated ALA auto-oxidation and increases the formation of superoxide, which is disproportionated to hydrogen peroxide and ALA in RBC, leading to oxidative damage in the RBC membrane. Moreover, Pb interferes with oxyhemoglobin, the main source of O_2_ in RBC, and increases ROS production, causing peroxidation damage to the RBC membrane and hemolysis [44].

Normally, oxidative damage induced by Pb metal activates antioxidant enzymes [20,27,39]. Some studies have shown that oxidation inhibition of ALA and Oxy HB occurs by superoxide dismutase (SOD) and catalase [20]. However, although some works have been conducted to investigate the oxidative effect of Pb on SOD activity, most of the results are not in consensus; reduced exposure to Pb by SOD has been reported in several clinical trials [20,42,43,45] and their studies, Patil et al. reported that the increase in serum malondialdehyde levels had increased linearly with increased lipid peroxidation due to increased levels of Pb blood [18,40]; in the present study, no significant effect was found between serum levels of Pb and its effect on the activity of MDA and SOD in the erythrocyte. Inconsistently with us, Zhang et al. reported that Lactobacillus fermentum HNU312 (Lf312) Lf312 by directly adsorbing lead ions in vivo modulated the structure and function of the gut microbiome, involved the upregulation of several metabolic pathways related to antioxidant, and promoted the release of SCFAs. Overall, Lf312 prevents oxidative damage and inflammatory responses induced by lead exposure [20].

Finally, no significant statistical differences in blood parameters and oxidative stress indices among study groups after six weeks. Most likely, this is an effect of the presence of metal cations such as Zinc (Zn), Iron (Fe), Calcium (Ca), and magnesium (Mg), which disrupt the process of removing Cd and Pb from aqueous solutions by two species of L. officinalis and B. longum, due to competition between these metals to bacterial binding sites, the binding capacity is reduced [36]. We postulate that a similar process may occur in our model organism and selected probiotics.

In this study, rats received continuous and prolonged periods of probiotic bacteria and were exposed daily to Pb. That was similar to regular human consumption of probiotics from food and exposure to lead. This method provides sufficient opportunity to introduce probiotics in the digestive system. Its extrapolation to humans is plausible, as it appears consistent with humans' natural process of exposure. Zhai et al. believe that colonizing bacteria within the digestive tract can reduce the number of free surfaces of Pb binding, enhancing its removal from the body [46]. In our work, this effect was not seen; a possible hypothesis is that the Pb detoxification process requires a longer interaction period, or the induction of lead acetate by gavage, or higher doses of it, or higher doses of bacteria were necessary to assess the Pb toxicity in rat models. Therefore, more studies are suggested, employing a greater number of rats, a longer period, and higher doses of Pb acetate and probiotics to precisely evaluate the effect of probiotics against Pb toxicity in rats. On the other hand, the accuracy of the blood lead level among the groups shows that the negative control group's blood lead level is higher than the positive control group's despite receiving lead in water. This issue shows that there may be unknown factors affecting lead absorption. Also, 54–78% of the lead coming out of the blood is excreted through the urine, and considering that the blood collection was started from this group, there is also a possibility that some blood lead from other groups may have been excreted in the urine. So, measuring the level of Urinary lead is also suggested for future studies.

## 5 Conclusions

Study results showed that administering probiotics or synbiotics fermented soy milk has no significant effect on blood parameters such as red blood cell counts, hematocrit and hemoglobin levels, and superoxide dismutase (SOD) activity and malondialdehyde (MDA) presence. More importantly, administering synbiotic fermented soybean milk containing L. acidophilus and inulin may significantly improve serum lead levels. However, laboratory studies suggest that probiotics may have the potential to remove heavy metals, although the exact effects in a living organism may differ due to several factors, such as the presence of co-occurring bacterial species, the gastrointestinal tract's pH, other metal cations or heavy metals, compounds in competition with metals (e.g., chelators), and the introduction of probiotics into the gastrointestinal tract. Nonetheless, this study presents a simple and effective method of preparing synbiotic food with potential chelation effects and provides an opportunity for further research and implementation in humans.

## Ethics approval

Animal experiments were approved and conducted in conformity with the Shiraz University of Medical Sciences Ethics Committee for the care and use of laboratory animals (Ethic No.: IR.SUMS.REC.1392.S6867).

## Author contribution statement

Maryamsadat Riasatian, Seyed Mohammad Mazloomi, Afsane Ahmadi: Conceived and designed the experiments; Performed the experiments; Analyzed and interpreted the data; Contributed reagents, materials, analysis tools or data; Wrote the paper. Zahra Derakhshan, Saeed Rajabi: Conceived and designed the experiments; Analyzed and interpreted the data; Contributed reagents, materials, analysis tools or data; Wrote the paper.

## Data availability statement

Data will be made available on request.

## Declaration of competing interest

The authors declare that they have no known competing financial interests or personal relationships that could have appeared to influence the work reported in this paper.
